# Hypoxia-Inducible Factor-2-Altered Urothelial Carcinoma: Clinical and Genomic Features

**DOI:** 10.3390/curroncol29110681

**Published:** 2022-11-14

**Authors:** Panagiotis J. Vlachostergios, Ioannis A. Tamposis, Maria Anagnostou, Maria Papathanassiou, Lampros Mitrakas, Ioannis Zachos, Eleni Thodou, Maria Samara, Vassilios Tzortzis

**Affiliations:** 1 Division of Hematology and Medical Oncology, Department of Medicine, Weill Cornell Medicine, New York, NY 10065, USA; 2 Department of Computer Science and Biomedical Informatics, University of Thessaly, 38221 Lamia, Greece; itamposis@uth.gr; 3 Department of Pathology and Cytology, University Hospital of Larissa, Faculty of Medicine, University of Thessaly, 41500 Larissa, Greece; managnostou@bio.uth.gr (M.A.); mpapat@uth.gr (M.P.); eleni.thodou@gmail.com (E.T.); msamar@uth.gr (M.S.); 4 Department of Urology, University Hospital of Larissa, Faculty of Medicine, University of Thessaly, 41500 Larissa, Greece; lmitrak@uth.gr (L.M.); johnbzac@yahoo.gr (I.Z.); tzorvas@otenet.gr (V.T.)

**Keywords:** hypoxia-inducible factor 2, *EPAS1*, urothelial carcinoma, bladder cancer, genomic landscape, prognosis, immune

## Abstract

*Background:* Hypoxia is recognized as a key feature of cancer growth and is involved in various cellular processes, including proliferation, angiogenesis, and immune surveillance. Besides hypoxia-inducible factor 1-alpha (HIF-1α), which is the main mediator of hypoxia effects and can also be activated under normoxic conditions, little is known about its counterpart, HIF-2. This study focused on investigating the clinical and molecular landscape of HIF-2-altered urothelial carcinoma (UC). *Methods:* Publicly available next-generation sequencing (NGS) data from muscle-invasive UC cell lines and patient tumor samples from the MSK/TCGA 2020 cohort (*n* = 476) were interrogated for the level of expression (mRNA, protein) and presence of mutations, copy number variations, structural variants in the *EPAS1* gene encoding HIF-2, and findings among various clinical (stage, grade, progression-free and overall survival) and molecular (tumor mutational burden, enriched gene expression) parameters were compared between altered and unaltered tumors. *Results:* 19% (7/37) of UC cell lines and 7% (27/380) of patients with muscle-invasive UC display high *EPAS1* mRNA and protein expression or/and EPAS1 alterations. *EPAS1*-altered tumors are associated with higher stage, grade, and lymph node metastasis as well as with shorter PFS (14 vs. 51 months, *q* = 0.01) and OS (15 vs. 55 months, *q* = 0.01). *EPAS1* mRNA expression is directly correlated with that of its target-genes, including *VEGF, FLT1, KDR, DLL4, CDH5, ANGPT1* (*q* < 0.001). While there is a slightly higher tumor mutational burden in *EPAS1*-altered tumors (9.9 vs. 4.9 mut/Mb), they are enriched in and associated with genes promoting immune evasion, including *ARID5B, SPINT1, AAK1, CLIC3, SORT1, SASH1*, and *FGFR3*, respectively (*q* < 0.001). *Conclusions*: HIF-2-altered UC has an aggressive clinical and a distinct genomic and immunogenomic profile enriched in angiogenesis- and immune evasion-promoting genes.

## 1. Introduction

Urothelial carcinoma is a lethal disease, particularly at advanced stages, yet it has seen unprecedented advances over the last five years. Novel therapeutic strategies, including immune checkpoint inhibitors (ICIs), antibody-drug conjugates (ADCs), and targeted therapies, for example against fibroblast growth factor receptor (FGFR)-mediated signaling, have entered the treatment paradigm and are leading to better patient outcomes [1,2,3,4,5]. Understanding who should get what treatment at what time, as well as predictive and prognostic biomarkers, will be key. Dissecting the molecular landscape of the disease may help identify particular subsets that could benefit from tailored therapies.

A major aspect in the development of various tumors, including UC, is growth under hypoxic or/and normoxic conditions that is orchestrated by activated signaling through two major transcription factors, hypoxia-inducible factor 1-alpha (HIF-1α) and HIF-2. High immunohistochemical (IHC) expression of HIF-1α in primary UC tumors is associated with higher-grade disease, vascular endothelial growth factor-related angiogenesis, and worse prognosis with regard to disease-free and overall survival in both superficial and invasive disease [6,7]. Hypoxia-induced autophagy may also propagate chemoresistance to cisplatin via the HIF-1α pathway [8]. Less is known about the role of HIF-2 and whether it may act as an oncogenic driver in UC.

This study aimed at unraveling the clinical and molecular landscape of HIF-2-altered UC by investigating publicly available next-generation sequencing (NGS) data from muscle-invasive UC cell lines and patient tumor samples.

## 2. Materials and Methods

The Cancer Cell Line Encyclopedia (CCLE) database [9] was used to query various primary cell lines for *EPAS1* mRNA and protein expression.

### 2.1. Patient Characteristics

A publicly available database, cBioportal for Cancer Genomics (www.cbioportal.org, accessed on 27 September 2022), was used to query DNA and RNA sequencing data for *EPAS1* mutations, copy number alterations, structural variants, mRNA and protein expression in a prospective multicenter cohort of 476 patients with muscle-invasive bladder UC (https://www.cbioportal.org/study/summary?id=blca_msk_tcga_2020, accessed on 25 October 2022).

The cohort included 334 patients analyzed via whole-exome sequencing and 142 patients analyzed via Memorial Sloan Kettering (MSK)-IMPACT sequencing. Sequencing was performed on fresh frozen or formalin-fixed paraffin-embedded specimens obtained via transurethral resection or RC. All patients were chemotherapy-naïve.

All computational analyses for exploring multidimensional cancer genomics data after integrating clinical profiles were performed as previously described [10,11].

### 2.2. Statistical Analyses

The Kaplan–Meier method was used to assess the association between altered and unaltered *EPAS1* with progression-free survival (PFS) and overall survival (OS), using a threshold z-score of ≥2.0. The Chi-squared and Kruskal-Wallis tests were used to compare clinical and pathological characteristics with altered vs. unaltered *EPAS1* tumors/patients. The student’s *t*-test was used to assess correlations between the expression of *EPAS1* and highly expressed genes in *EPAS1* altered vs. unaltered tumors. The Spearman’s correlation test was used to measure the degree of association between the expression of selected genes and *EPAS1*. Multiple hypothesis test correction was applied using the Benjamini–Hochberg method. *p* and *q* values of <0.05 were considered significant for all analyses.

## 3. Results

### 3.1. EPAS1 Expression in UC Cancer Cell Lines

**Figure 1:**
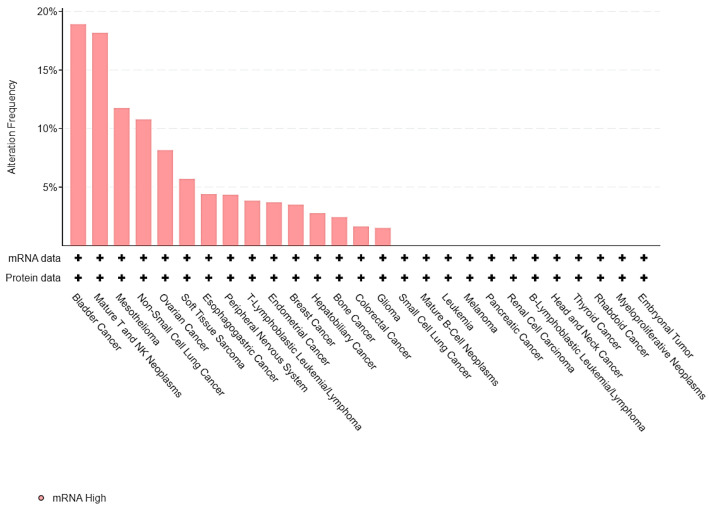
Barplot of EPAS1 expression across different cell lines from the Cancer Cell Line Encyclopedia (CCLE).

To assess the expression of HIF-2 in UC relative to various cancer types, the Cancer Cell Line Encyclopedia (CCLE) [9] was interrogated for mRNA and protein levels of EPAS1, the gene encoding HIF-2 protein. Seven out of 37 bladder UC cases (19%) displayed high EPAS1 expression, which was the highest proportion among all different cell types (Figure 1).

Thus, HIF-2 is overexpressed in up to one-fifth of UC cancer cells and could represent another mechanism of UC proliferation and growth.

### 3.2. EPAS1 Genomic Alterations in UC Patients

**Figure 2:**
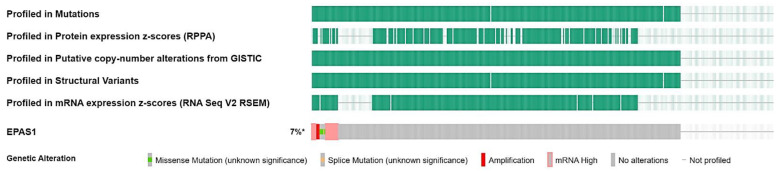
Oncoplot of *EPAS1* molecular alterations in MSK/TCGA 2020 cohort (*n* = 476). * altered/profiled: 27/380.

To dissect the molecular landscape of *EPAS1* molecular alterations in UC, publicly available data from 476 patients from MSK/TCGA 2020 were analyzed through cbioportal.org. EPAS1 was altered in 27 (7%) of patients-samples (Figure 2).

**Figure 3:**
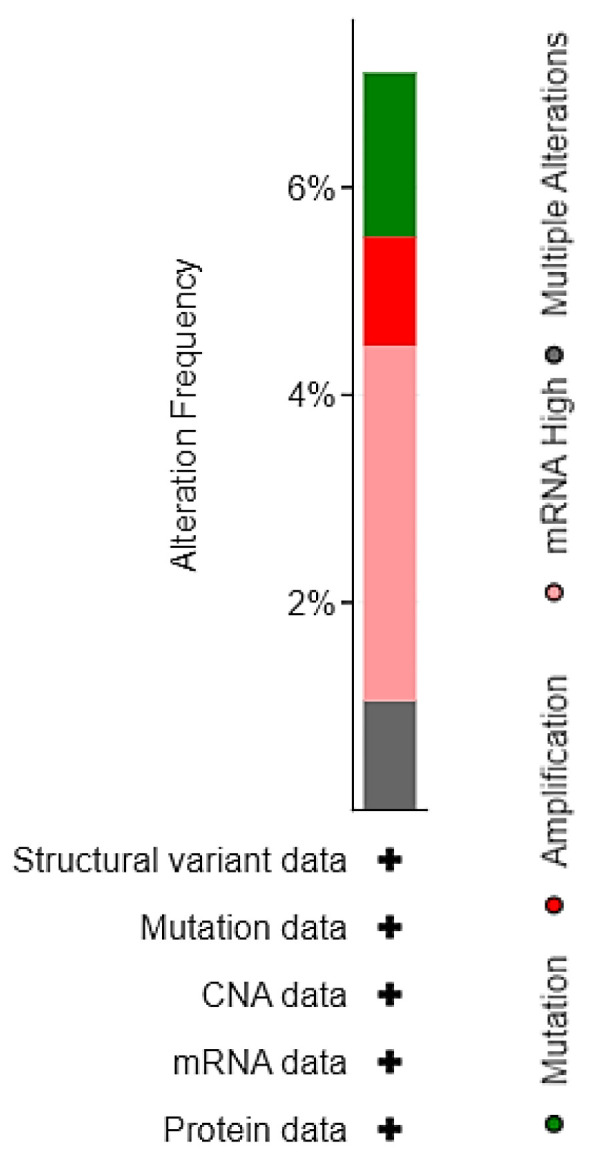
Distribution of EPAS1 molecular alterations in MSK/TCGA 2020 cohort (*n* = 476).

Most alterations involved high transcript levels of EPAS1 in 13 cases (3.5%), followed by mutations (1.6%), and amplification (1%), while another four patients (1%) had multiple alterations (Figure 3).

**Figure 4:**
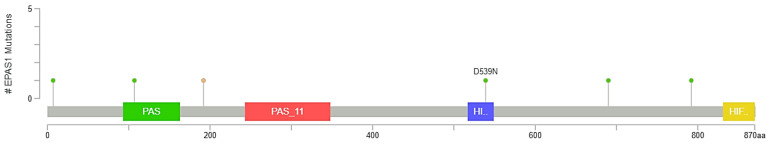
Lollipop plot of *EPAS1* mutations in MSK/TCGA 2020 cohort (*n* = 476). # Number of mutations.

There were six mutations in total, of which five were missense and one splice (Table 1, Figure 4).

### 3.3. Prognostic Value of EPAS1 Alterations in UC Patients

**Figure 5:**
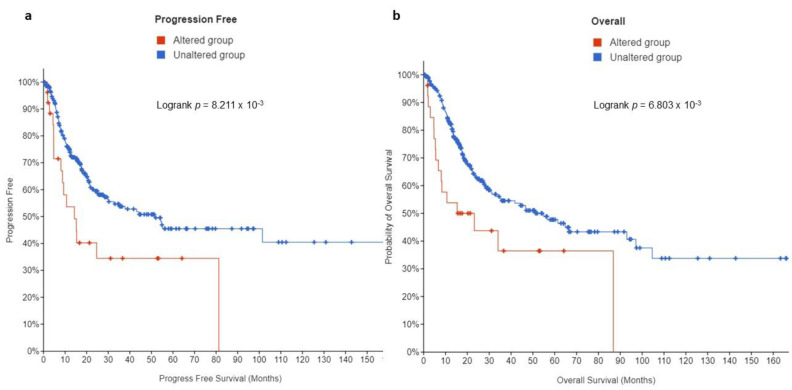
Kaplan Meier plots for (**a**) PFS and (**b**) OS in muscle-invasive UC patients from the MSK/TCGA 2020 cohort (*n* = 476).

This study then sought to examine whether there is a prognostic significance of *EPAS1*-altered UC in patients with muscle-invasive bladder UC. Kaplan-Meier analysis revealed that patients whose tumors displayed *EPAS1* overexpression had a significantly shorter progression-free survival (PFS) (14 months) compared to those without alterations (51 months, *q*-value = 0.0123) (Figure 5a). Likewise, high *EPAS1* expression predicted a shorter overall survival (OS, 15 vs. 55 months, *q*-value = 0.0123) (Figure 5b).

### 3.4. Clinical and Molecular Characteristics of HIF-2-Altered UC

**Figure 6:**
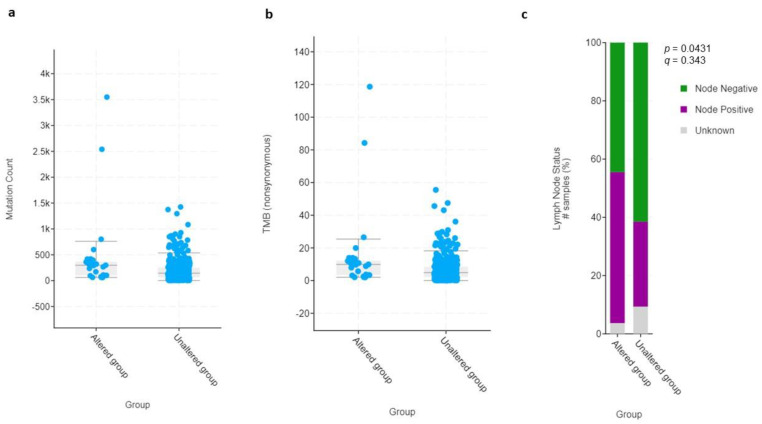
Dot plot graphs of (**a**) mutation count and (**b**) total mutational burden (TMB) of HIF-2-altered and unaltered tumors. (**c**) Bar graph of lymph node status of HIF-2-altered and unaltered tumors. # Number of samples (%).

To investigate any specific phenotypic/genotypic pattern in the subset of patients with HIF-2-altered UC, we compared the most common clinical, pathological, and molecular characteristics with those that did not demonstrate any HIF-2 alterations. The two groups were equally distributed with respect to age, sex, and race (Figure S1). There were 325 patients in total who were first diagnosed with primary muscle-invasive bladder cancer (MIBC), whereas 55 had progressed to T2 status (secondary MIBC) after an initial diagnosis of non-MIBC (Figure S1). The median number of mutations (298 vs. 144), as well as the total mutational burden (TMB) (9.9 vs. 4.9 mut/Mb) and nodal involvement (52% vs. 29%), were proportionately, but not significantly, more frequent in patients with tumors harboring high HIF-2 expression compared to those without HIF-2 alterations (Figure 6).

**Figure 7:**
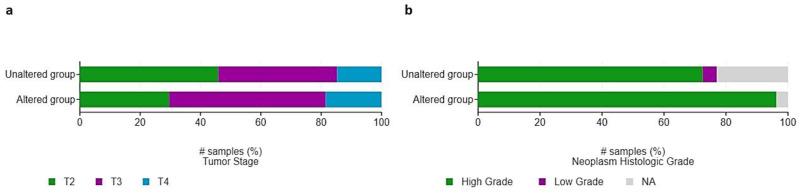
Distribution of (**a**) pathological T-stage and (**b**) grade among patients with HIF-2-altered and unaltered tumors. # Number of samples (%).

Additionally, there was a proportionally higher frequency of larger and high-grade tumors (T3: 52%; T4: 19%; high-grade: 96%) within the HIF-2-altered group compared to the unaltered group (T3: 39%; T4: 15%; high-grade: 72%); however, this did not reach the level of statistical significance (Figure 7). Fifty-five patients, of whom five with HIF-2-altered and 50 with non-altered tumors had received prior intravesical Bacillus Calmette-Guerin (BCG). There were no significant associations between prior intravesical therapy with BCG, or radiation therapy, and the presence of HIF-2 alterations (*q*-values > 0.05).

Gene-enrichment analysis in HIF-2-altered UC revealed interesting correlations with high ARID5B, SPINT1, AAK1, CLIC3, SORT1, and SASH1expression within a 15-gene list (Table 2).

### 3.5. EPAS1 Is Directly Associated with Expression of HIF-2-Target Genes

**Figure 8:**
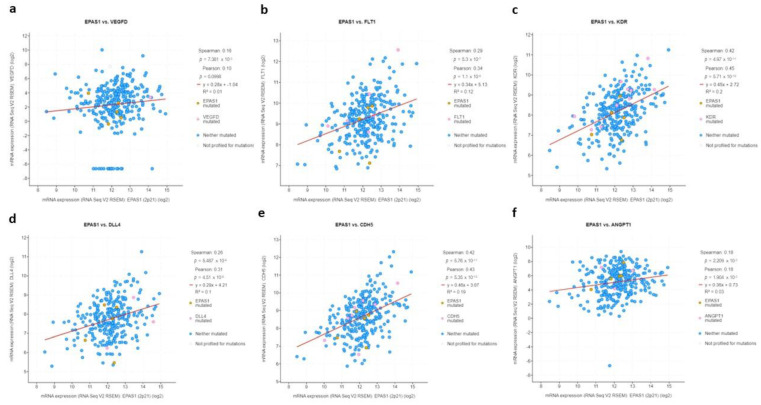
mRNA log2 expression of HIF-2-regulated genes (**a**) *VEGFD*, (**b**) *FLT1*, (**c**) *KDR*, (**d**) *DLL4*, (**e**) *CDH5*, (**f**) *ANGPT1* in association with *EPAS1* expression.

To functionally assess whether HIF-2 is transcriptionally active and mediates proliferation and angiogenic signals in HIF-2-altered tumors, mRNA levels of HIF-2-regulated genes, involved in various aspects of angiogenesis [12,13,14] were examined. Indeed, mRNA levels of *VEGFD*, *FLT1*, *KDR*, *DLL4*, *CDH5*, and *ANGPT1* were significantly and directly associated with EPAS1 mRNA expression (Figure 8).

### 3.6. EPAS1 Is Associated with Expression of Immune Suppression Genes

Since UC is known to be immunogenic and responsive to immunotherapy including intravesical BCG and ICIs, this study sought to investigate whether this “aggressive” and poor prognosis subset of HIF-2-altered tumors could be associated with aberrant expression of key genes involved in regulating immune responses in UC. Based on previous works from others and ours [15,16,17], *FGFR3* and *IFNG* genes were chosen, and their mRNA expression was assessed in association with *EPAS1*.

**Figure 9:**
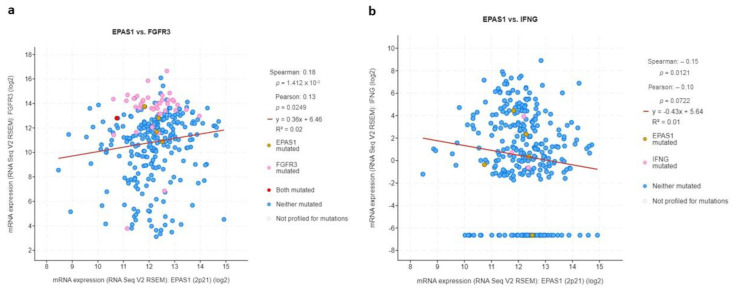
mRNA log2 expression of immune-modulating genes (**a**) *FGFR3* and (**b**) *IFNG* in association with *EPAS1* expression.

In accordance with their opposite biological roles in this context, *FGFR3* was directly associated with *EPAS1* expression (*r* = 0.18, *q*-value = 0.01) whereas *IFNG* was inversely correlated with *EPAS1* (*r* = −0.15, *q*-value = 0.05) (Figure 9).

## 4. Discussion

This study examined the significance of HIF-2 in UC. First, by exploring HIF-2 mRNA and protein expression among several different cell types, it provides evidence that bladder UC is the most commonly affected tumor type with the highest frequency of HIF-2/*EPAS1* overexpression. Second, by using the largest-to-date cohort of muscle-invasive UC this study revealed a distinct molecular and clinical profile in a subset of patients carrying somatic alterations in HIF-2/*EPAS1*.While these occur in nearly one-tenth of patients/tumors, they are associated with more aggressive histopathological features including higher T stage, presence of adenopathy, and high-grade disease. The presence of *EPAS1* alterations, the majority of which involves high *EPAS1* expression and/or amplification, is predictive of shorter PFS and OS. At the molecular level, HIF-2-altered UC tumors display a direct association between *EPAS1* mRNA and expression of its target-genes, including *VEGF*, *FLT1*, *KDR*, *DLL4*, *CDH5*, and *ANGPT1*. While there is a slightly higher overall mutation burden in *EPAS1*-altered tumors, they are enriched in and associated with genes promoting immune evasion, including *ARID5B, SPINT1*, *AAK1, CLIC3, SORT1, SASH1,* and *FGFR3*, respectively.

Expression of HIF-2/*EPAS1* in bladder UC cell lines and a small number of paraffin-embedded samples from patients has been previously reported to be more abundant in muscle-invasive compared to superficial disease [18]. Interestingly, HIF-2 protein was not found in cancer cells or in normal tissues but rather in stroma around cancer cells, particularly in tumor-associated macrophages (TAMs) within perinecrotic regions, whereby it correlated with higher pathological stage, grade, and VEGF-related tumor angiogenesis [19]. Moreover, patients with tumors harboring *EPAS1*-expressing TAMs were characterized by shorter cancer-specific survival [20].

This study, by providing a more comprehensive assessment of HIF-2 genomic alterations at the DNA, RNA, and protein level, confirms and complements previous preliminary evidence, supporting a negative prognostic role of HIF-2/EPAS1 expression on both PFS and OS. More importantly, this study describes for the first time the molecular landscape of HIF-2-altered muscle-invasive UC. It shows that HIF-2-overexpressing or/and amplified UC tumors are characterized by active angiogenic signaling, evidenced by the direct association of expression of *EPAS1* with HIF-2-regulated genes involved in the initiation of angiogenesis (*VEGF, FLT1, KDR, CDH5, DLL4*), neo-vessel formation (*VEGF, FLT1, KDR, CDH5, DLL4*) and maturation (*VEGF, ANGPT1*) [11]. Further, these HIF-2-altered tumors demonstrate a slightly higher but not significantly different mutational load compared to UC lacking HIF-2 alterations. Thus, while an overall TMB of above 10 is usually predictive of response to ICIs in various primaries [21], HIF-2-overexpressing tumors behave rather in the opposite direction due to overexpression of *ARID5B*, *SPINT1, AAK1, CLIC3, SORT1,* and *SASH1*.

The AT-rich interaction domain (ARID) family is a superfamily belonging to switch/sucrose nonfermenting (SWI/SNF) chromatin remodeling complexes and the presence of inactivating mutations in any of their members, including ARID5B, has been associated with greater benefit from ICI therapy in pan-cancer analyses [22]. Conversely, the enrichment of *ARID5B* expression in HIF-2-altered tumors could suggest innate resistance to ICIs. SPINT1, also known as hepatocyte growth factor activator inhibitor 1 (HAI-1) is an endogenous protease inhibitor of HGF that is found at increased levels in tumors and urine from patients with muscle-invasive UC [23] and may be involved in migration and metastasis [24]. The finding of enhanced *SPINT1* expression in HIF-2-altered tumors could also have an impact on these tumors’ resistance to ICIs in view of recent data including *SPINT1* in an 8-gene prognostic signature that stratifies patients with bladder UC into two risk groups with distinct immune profile and responsiveness to immunotherapy [25]. AAK1 is involved in clathrin-mediated endocytosis of chemokine receptors and AAK1-inhibition in mice resulted in increased intratumoral infiltration, supporting an immune-suppressive role for this gene product, as well [26]. CLIC3 is a chloride intracellular channel protein the gene expression of which has been correlated with low immune infiltration of myeloid dendritic cells (DCs) and poor prognosis in hepatocellular carcinoma [27]. Therefore, it is possible that a similar immune suppressive role could be exerted in HIF-2-altered UC. *SORT1* is another gene enriched in HIF-2 amplified UC tumors that are likely to be involved in allowing cancer cells to evade the immune system, given previously reported negative correlation with the infiltration levels of DCs, cytotoxic T cells, NK CD56dim cells, Tgd, and pDCs, as well as a positive correlation with immune evasion checkpoints including *PDCD1*, *CD274*, and *CTLA-4* [28]. *SASH1* encodes a scaffold molecule involved in NF-kappa-B activation and promotes immune escape via subsequent upregulation of PD-L1 expression [29,30].

In further support of this immune-suppressive environment forged by HIF-2 overexpression in UC is the presence of a direct association between *EPAS1* and *FGFR3* mRNA levels while the opposite was the case between *EPAS1* and *IFNG* expression. FGFR3, as previously described by many groups, including ours, is a major player causing immune resistance in both lower and upper tract UC which is often associated with attenuated IFNG signaling [17]. In presence of an approved therapy for advanced UC, erdafitinib [5], FGFR3-inhibition could become a valid strategy to overcome the “immune-cold” nature of HIF-2-altered UC. Besides that, specific HIF-2 inhibitors, such as belzutifan, are already designed and in an advanced phase of testing in renal cell carcinoma, with promising activity [31]. Stromal HIF-2 exerts an immune suppressive role in other tumor types as well, for example, pancreatic adenocarcinoma [32]. By revealing a network of genes-players that are involved in this process in direct association with *HIF-2* in UC, this study provides several insights for deepening our understanding of the mechanistic processes that are potentially involved.

The rest group of genes that were significantly upregulated in HIF-2-altered tumors are involved in urothelial differentiation (*UPK2*) [33], stemness (*GPR78, HS3ST2*) [34,35], epithelial-mesenchymal transition (CRYBG2) [36], PI3K/Akt/beta-catenin signaling (*VGLL1*) [37], MAPK signaling (SH3TC2, GAREM1) [38,39], coactivation of different nuclear receptors (*NCOA1*) [40], ER stress and hedgehog signaling (*CREB3L2*) [41], TGF-beta and WNT signaling (CRYBG2) [36].

This study was limited by its computational design and single cohort evaluation, which is however the largest reported-to-date with both molecular and survival data available. This work represents the first comprehensive effort to study the molecular and clinical significance of HIF-2-altered UC. This study’s findings place UC within a particular group of cancers, including renal cell carcinoma, HER2-positive breast cancer, hepatocellular carcinoma, and head and neck cancers, in which HIF-2/EPAS1 expression has prognostic value [42,43,44,45,46]. Additional studies are needed to further elucidate the genomic and immunogenomic profile of HIF-2-altered UC in order to design targeted therapies for this small but important subset of patients.

## Figures and Tables

**Table 1:** *EPAS1* mutations in MSK/TCGA 2020 cohort (*n* = 476). # Total number of nonsynonymous mutations.

Sample ID	Protein Change	Mutation Type	Allele Freq (T)	# Mut in Sample
MSKCC-0450_NR	D539N	Missense	NA	61
TCGA-DK-A1A5-01	R690Q	Missense	0.25	236
TCGA-E5-A4TZ-01	D107N	Missense	0.16	420
TCGA-FD-A5BV-01	K7R	Missense	0.35	111
TCGA-S5-A6DX-01	X192_splice	Splice	0.27	600
TCGA-UY-A78N-01	G792R	Missense	0.22	267

**Table 2:** mRNA expression of genes enriched in HIF-2-altered tumors. μ: mean log2 gene expression, σ: standard deviation of log2 gene expression.

Gene	Cytoband	μ in Altered Group	μ in Unaltered Group	σ in Altered Group	σ in Unaltered Group	Log Ratio	*p*-Value	*q*-Value
*VGLL1*	Xq26.3	11.01	9.07	1.4	2.85	1.94	2.8 × 10^−7^	6.996 × 10^−4^
*UPK2*	11q23.3	12.59	9.78	2.44	4.23	2.8	6.397 × 10^−6^	7.116 × 10^−3^
*EPAS1*	2p21	13.4	12.04	1.31	0.91	1.35	1.979 × 10^−5^	0.0152
*GPR78*	4p16.1	5.77	3.92	1.77	2.81	1.85	2.527 × 10^−5^	0.0158
*SPINT1*	15q15.1	13.35	12.6	0.73	1.1	0.74	3.785 × 10^−5^	0.0205
*HS3ST2*	16p12.2	4.72	3.42	1.3	1.89	1.3	4.418 × 10^−5^	0.0233
*SH3TC2*	5q32	8.3	7.49	0.79	1.7	0.81	6.16 × 10^−5^	0.0268
*GAREM1*	18q12.1	8.61	7.85	0.78	1.3	0.76	7.524 × 10^−5^	0.0284
*AAK1*	2p13.3	10.46	9.99	0.51	0.56	0.47	8.999 × 10^−5^	0.0328
*NCOA1*	2p23.3	10.51	9.99	0.57	0.65	0.53	9.475 × 10^−5^	0.0339
*CLIC3*	9q34.3	10.15	8.25	2.06	2.54	1.9	1.105 × 10^−4^	0.0382
*ARID5B*	10q21.2	10.61	10.02	0.64	0.89	0.59	1.273 × 10^−4^	0.0404
*CREB3L2*	7q33	11.25	10.57	0.76	0.93	0.68	1.446 × 10^−4^	0.0419
*SORT1*	1p13.3	11.18	10.55	0.69	0.94	0.63	1.528 × 10^−4^	0.0419
*SASH1*	6q24.3-q25.1	10.15	9.34	0.91	0.93	0.81	1.541 × 10^−4^	0.0419
*CRYBG2*	1p36.11	9.72	8.66	1.19	1.74	1.06	1.86 × 10^−4^	0.0481

## Data Availability

All data used for this analysis are available at https://www.cbioportal.org/study/summary?id=blca_msk_tcga_2020 (accessed on 25 October 2022).

## Supplementary Materials

The following supporting information can be downloaded at: https://www.mdpi.com/1718-7729/29/11/681/s1, Figure S1: Clinico-pathological characteristics without significant differences between EPAS-altered and unaltered tumors. (a) Dot plot graphs of Diagnosis Age of EPAS-altered and un-altered tumors; Bar graph of (b) Race Category; (c) Sex; (d) Primary vs. Secondary; (e) Prior Intravesical Chemotherapy of EPAS-altered and un-altered tumors. ^#^ Number of samples (%).

## References

[1] Bellmunt J., de Wit R., Vaughn D.J., Fradet Y., Lee J.L., Fong L., Vogelzang N.J., Climent M.A., Petrylak D.P., Choueiri T.K. (2017). Pembrolizumab as Second-Line Therapy for Advanced Urothelial Carcinoma. N. Engl. J. Med..

[2] Powles T., Durán I., van der Heijden M.S., Loriot Y., Vogelzang N.J., De Giorgi U., Oudard S., Retz M.M., Castellano D., Bamias A. (2018). Atezolizumab versus chemotherapy in patients with platinum-treated locally advanced or metastatic urothelial carcinoma (IMvigor211): A multicentre, open-label, phase 3 randomised controlled trial. Lancet.

[3] Rosenberg J.E., O’Donnell P.H., Balar A.V., McGregor B.A., Heath E.I., Yu E.Y., Galsky M.D., Hahn N.M., Gartner E.M., Pinelli J.M. (2019). Pivotal Trial of Enfortumab Vedotin in Urothelial Carcinoma after Platinum and Anti-Programmed Death 1/Programmed Death Ligand 1 Therapy. J. Clin. Oncol..

[4] Powles T., Rosenberg J.E., Sonpavde G.P., Loriot Y., Durán I., Lee J.L., Matsubara N., Vulsteke C., Castellano D., Wu C. (2021). Enfortumab Vedotin in Previously Treated Advanced Urothelial Carcinoma. N. Engl. J. Med..

[5] Loriot Y., Necchi A., Park S.H., Garcia-Donas J., Huddart R., Burgess E., Fleming M., Rezazadeh A., Mellado B., Varlamov S. (2019). Erdafitinib in Locally Advanced or Metastatic Urothelial Carcinoma. N. Engl. J. Med..

[6] Theodoropoulos V.E., Lazaris A.C., Sofras F., Gerzelis I., Tsoukala V., Ghikonti I., Manikas K., Kastriotis I. (2004). Hypoxia-inducible factor 1 alpha expression correlates with angiogenesis and unfavorable prognosis in bladder cancer. Eur. Urol..

[7] Palit V., Phillips R.M., Puri R., Shah T., Bibby M.C. (2005). Expression of HIF-1alpha and Glut-1 in human bladder cancer. Oncol. Rep..

[8] Mao X., Nanzhang U., Xiao J., Wu H., Ding K. (2021). Hypoxia-Induced Autophagy Enhances Cisplatin Resistance in Human Bladder Cancer Cells by Targeting Hypoxia-Inducible Factor-1α. J. Immunol. Res..

[9] Ghandi M., Huang F.W., Jané-Valbuena J., Kryukov G.V., Lo C.C., McDonald E.R., Barretina J., Gelfand E.T., Bielski C.M., Li H. (2019). Next-generation characterization of the Cancer Cell Line Encyclopedia. Nature.

[10] Cerami E., Gao J., Dogrusoz U., Gross B.E., Sumer S.O., Aksoy B.A., Jacobsen A., Byrne C.J., Heuer M.L., Larsson E. (2012). The cBio cancer genomics portal: An open platform for exploring multidimensional cancer genomics data. Cancer Discov..

[11] Gao J., Aksoy B.A., Dogrusoz U., Dresdner G., Gross B., Sumer S.O., Sun Y., Jacobsen A., Sinha R., Larsson E. (2013). Integrative analysis of complex cancer genomics and clinical profiles using the cBioPortal. Sci. Signal..

[12] Jones A., Fujiyama C., Blanche C., Moore J.W., Fuggle S., Cranston D., Bicknell R., Harris A.L. (2001). Relation of vascular endothelial growth factor production to expression and regulation of hypoxia-inducible factor-1 alpha and hypoxia-inducible factor-2 alpha in human bladder tumors and cell lines. Clin. Cancer Res..

[13] Befani C., Liakos P. (2018). The role of hypoxia-inducible factor-2 alpha in angiogenesis. J. Cell Physiol..

[14] Hu C.J., Wang L.Y., Chodosh L.A., Keith B., Simon M.C. (2003). Differential roles of hypoxia-inducible factor 1alpha (HIF-1alpha) and HIF-2alpha in hypoxic gene regulation. Mol. Cell Biol..

[15] Cathomas R., Lorch A., Bruins H.M., Compérat E.M., Cowan N.C., Efstathiou J.A., Fietkau R., Gakis G., Hernández V., Espinós E.L. (2022). The 2021 Updated European Association of Urology Guidelines on Metastatic Urothelial Carcinoma. Eur. Urol..

[16] Chen S., Zhang N., Shao J., Wang T., Wang X. (2019). Multi-omics Perspective on the Tumor Microenvironment based on PD-L1 and CD8 T-Cell Infiltration in Urothelial Cancer. J. Cancer.

[17] Robinson B.D., Vlachostergios P.J., Bhinder B., Liu W., Li K., Moss T.J., Bareja R., Park K., Tavassoli P., Cyrta J. (2019). Upper tract urothelial carcinoma has a luminal-papillary T-cell depleted contexture and activated FGFR3 signaling. Nat. Commun..

[18] Xia G., Kageyama Y., Hayashi T., Hyochi N., Kawakami S., Kihara K. (2002). Positive expression of HIF-2alpha/EPAS1 in invasive bladder cancer. Urology.

[19] Onita T., Ji P.G., Xuan J.W., Sakai H., Kanetake H., Maxwell P.H., Fong G.H., Gabril M.Y., Moussa M., Chin J.L. (2002). Hypoxia-induced, perinecrotic expression of endothelial Per-ARNT-Sim domain protein-1/hypoxia-inducible factor-2alpha correlates with tumor progression, vascularization, and focal macrophage infiltration in bladder cancer. Clin. Cancer Res..

[20] Koga F., Kageyama Y., Kawakami S., Fujii Y., Hyochi N., Ando N., Takizawa T., Saito K., Iwai A., Masuda H. (2004). Prognostic significance of endothelial Per-Arnt-sim domain protein 1/hypoxia-inducible factor-2alpha expression in a subset of tumor associated macrophages in invasive bladder cancer. J. Urol..

[21] Marabelle A., Fakih M., Lopez J., Shah M., Shapira-Frommer R., Nakagawa K., Chung H.C., Kindler H.L., Lopez-Martin J.A., Miller W.H. (2020). Association of tumour mutational burden with outcomes in patients with advanced solid tumours treated with pembrolizumab: Prospective biomarker analysis of the multicohort, open-label, phase 2 KEYNOTE-158 study. Lancet Oncol..

[22] Zhu Y., Yan C., Wang X., Xu Z., Lv J., Xu X., Yu W., Zhou M., Yue L. (2022). Pan-cancer analysis of ARID family members as novel biomarkers for immune checkpoint inhibitor therapy. Cancer Biol. Ther..

[23] Shimwell N.J., Bryan R.T., Wei W., James N.D., Cheng K.K., Zeegers M.P., Johnson P.J., Martin A., Ward D.G. (2013). Combined proteome and transcriptome analyses for the discovery of urinary biomarkers for urothelial carcinoma. Br. J. Cancer.

[24] Chen Y., Xu T., Xie F., Wang L., Liang Z., Li D., Liang Y., Zhao K., Qi X., Yang X. (2021). Evaluating the biological functions of the prognostic genes identified by the Pathology Atlas in bladder cancer. Oncol. Rep..

[25] Zhang Y., Lin Y., Lv D., Wu X., Li W., Wang X., Jiang D. (2022). Identification and validation of a novel signature for prediction the prognosis and immunotherapy benefit in bladder cancer. PeerJ.

[26] Rogers L.M., Wang Z., Mott S.L., Dupuy A.J., Weiner G.J. (2020). A Genetic Screen to Identify Gain- and Loss-of-Function Modifications that Enhance T-cell Infiltration into Tumors. Cancer Immunol. Res..

[27] Huang J.J., Lin J., Chen X., Zhu W. (2021). Identification of chloride intracellular channels as prognostic factors correlated with immune infiltration in hepatocellular carcinoma using bioinformatics analysis. Medicine.

[28] Gao Y., Li Y., Song Z., Jin Z., Li X., Yuan C. (2022). Sortilin 1 Promotes Hepatocellular Carcinoma Cell Proliferation and Migration by Regulating Immune Cell Infiltration. J. Oncol..

[29] Shi L., Yang Y., Li M., Li C., Zhou Z., Tang G., Wu L., Yao Y., Shen X., Hou Z. (2022). LncRNA IFITM4P promotes immune escape by up-regulating PD-L1 via dual mechanism in oral carcinogenesis. Mol. Ther..

[30] Antonangeli F., Natalini A., Garassino M.C., Sica A., Santoni A., Di Rosa F. (2020). Regulation of PD-L1 Expression by NF-κB in Cancer. Front. Immunol..

[31] Fallah J., Brave M.H., Weinstock C., Mehta G.U., Bradford D., Gittleman H., Bloomquist E.W., Charlab R., Hamed S.S., Miller C.P. (2022). FDA Approval Summary: Belzutifan for von Hippel-Lindau disease associated tumors. Clin. Cancer Res..

[32] Garcia Garcia C.J., Huang Y., Fuentes N.R., Turner M.C., Monberg M.E., Lin D., Nguyen N.D., Fujimoto T.N., Zhao J., Lee J.J. (2022). Stromal HIF2 Regulates Immune Suppression in the Pancreatic Cancer Microenvironment. Gastroenterology.

[33] Boumelhem B.B., Fraser S.T., Assinder S.J. (2019). Differentiation of Urothelium from Mouse Embryonic Stem Cells in Chemically Defined Conditions. Methods Mol. Biol..

[34] Yan Y., He M., Zhao L., Wu H., Zhao Y., Han L., Wei B., Ye D., Lv X., Wang Y. (2022). A novel HIF-2α targeted inhibitor suppresses hypoxia-induced breast cancer stemness via SOD2-mtROS-PDI/GPR78-UPRER axis. Cell Death Differ..

[35] Teixeira F.C.O.B., Vijaya Kumar A., Kumar Katakam S., Cocola C., Pelucchi P., Graf M., Kiesel L., Reinbold R., Pavão M.S.G., Greve B. (2020). The Heparan Sulfate Sulfotransferases HS2ST1 and HS3ST2 Are Novel Regulators of Breast Cancer Stem-Cell Properties. Front. Cell Dev. Biol..

[36] Li M., Liu S., Huang W., Zhang J. (2021). Physiological and pathological functions of βB2-crystallins in multiple organs: A systematic review. Aging.

[37] Kim B.K., Cheong J.H., Im J.Y., Ban H.S., Kim S.K., Kang M.J., Lee J., Kim S.Y., Park K.C., Paik S. (2019). PI3K/AKT/β-Catenin Signaling Regulates Vestigial-Like 1 Which Predicts Poor Prognosis and Enhances Malignant Phenotype in Gastric Cancer. Cancers.

[38] Huang C., Yi H., Zhou Y., Zhang Q., Yao X. (2022). Pan-Cancer Analysis Reveals SH3TC2 as an Oncogene for Colorectal Cancer and Promotes Tumorigenesis via the MAPK Pathway. Cancers.

[39] Taniguchi T., Tanaka S., Ishii A., Watanabe M., Fujitani N., Sugeo A., Gotoh S., Ohta T., Hiyoshi M., Matsuzaki H. (2013). A brain-specific Grb2-associated regulator of extracellular signal-regulated kinase (Erk)/mitogen-activated protein kinase (MAPK) (GAREM) subtype, GAREM2, contributes to neurite outgrowth of neuroblastoma cells by regulating Erk signaling. J. Biol. Chem..

[40] Triki M., Lapierre M., Cavailles V., Mokdad-Gargouri R. (2017). Expression and role of nuclear receptor coregulators in colorectal cancer. World J. Gastroenterol..

[41] Iwamoto H., Matsuhisa K., Saito A., Kanemoto S., Asada R., Hino K., Takai T., Cui M., Cui X., Kaneko M. (2015). Promotion of Cancer Cell Proliferation by Cleaved and Secreted Luminal Domains of ER Stress Transducer BBF2H7. PLoS ONE.

[42] Chen B., Wang L., Zhao J., Tan C., Zhao P. (2021). Expression and prognostic significance of EPAS-1 in renal clear cell carcinoma. Ann. Ital. Chir..

[43] Jarman E.J., Ward C., Turnbull A.K., Martinez-Perez C., Meehan J., Xintaropoulou C., Sims A.H., Langdon S.P. (2019). HER2 regulates HIF-2α and drives an increased hypoxic response in breast cancer. Breast Cancer Res..

[44] Eskiizmir G., Çalıbaşı Koçal G., Uysal T., Ellidokuz H., Başpınar Y. (2021). Serum hypoxia-inducible factor-2: A candidate prognostic biomarker for laryngeal cancer. Clin. Otolaryngol..

[45] Lim E., Kuo C.C., Tu H.F., Yang C.C. (2017). The prognosis outcome of oral squamous cell carcinoma using HIF-2α. J. Chin. Med. Assoc..

[46] Bangoura G., Liu Z.S., Qian Q., Jiang C.Q., Yang G.F., Jing S. (2007). Prognostic significance of HIF-2alpha/EPAS1 expression in hepatocellular carcinoma. World J. Gastroenterol..

