# Supplementary material for: Hypoxia-Inducible Factor-2-Altered Urothelial Carcinoma: Clinical and Genomic Features
Source: Curr Oncol. 2022 Nov 14;29(11):8638–49. doi: 10.3390/curroncol29110681 (PMC9689673; doi:10.3390/curroncol29110681)
Supplement: Supplementary file 1 [file curroncol-29-00681-s001.zip › curroncol-2022694-supplementary.pdf]

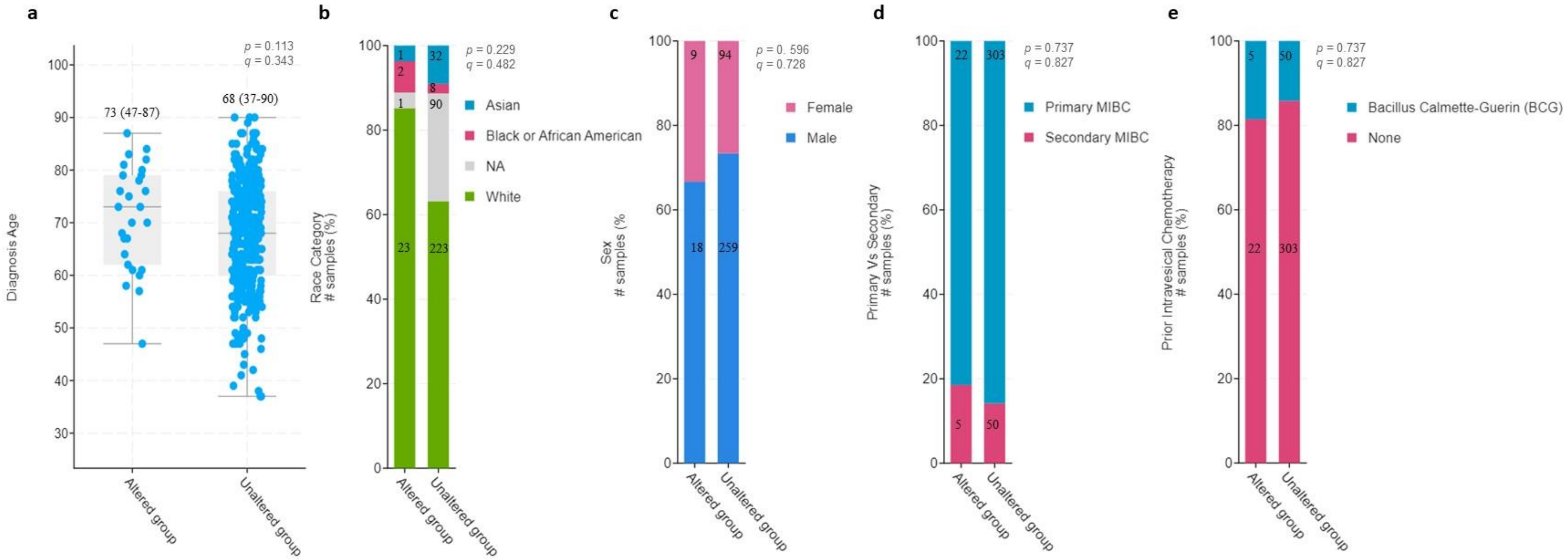

Figure S1: Clinico-pathological characteristics without significant differences between EPAS-altered and unaltered tumors. (a) Dot plot graphs of Diagnosis Age of EPAS-altered and un-altered tumors; Bar graph of (b) Race Category; (c) Sex; (d) Primary vs. Secondary; (e) Prior Intravesical Chemotherapy of EPAS-altered and un-altered tumors. # Number of samples (%).
